# Multimodal Photoacoustic Imaging‐Guided Regression of Corneal Neovascularization: A Non‐Invasive and Safe Strategy

**DOI:** 10.1002/advs.202000346

**Published:** 2020-05-29

**Authors:** Chengchao Chu, Jingwen Yu, En Ren, Shangkun Ou, Yunming Zhang, Yiming Wu, Han Wu, Yang Zhang, Jing Zhu, Qixuan Dai, Xiaoyong Wang, Qingliang Zhao, Wei Li, Zuguo Liu, Xiaoyuan Chen, Gang Liu

**Affiliations:** ^1^ State Key Laboratory of Molecular Vaccinology and Molecular Diagnostics & Center for Molecular Imaging and Translational Medicine School of Public Health Xiamen University Xiamen 361102 China; ^2^ Fujian Provincial Key Laboratory of Ophthalmology and Visual Science School of Medicine Xiamen University Xiamen 361102 China; ^3^ Laboratory of Molecular Imaging and Nanomedicine National Institute of Biomedical Imaging and Bioengineering (NIBIB) National Institutes of Health (NIH) Bethesda MD 20892 USA

**Keywords:** corneal neovascularization, eye drops, gene therapy, photoacoustic imaging, phototherapy

## Abstract

Corneal neovascularization (CNV) is one of the main factors that induce blindness worldwide. However, current medical treatments cannot achieve non‐invasive and safe inhibition of CNV. A noninvasive photoacoustic imaging (PAI)‐guided method is purposed for the regression of CNV. PAI can monitor the oxygen saturation of cornea blood vessels through the endogenous contrast of hemoglobin and trace administrated drugs by themselves as exogenous contrast agents. An indocyanine green (ICG)‐based nanocomposite (R‐s‐ICG) is prepared for CNV treatment via eye drops and subconjunctival injections. It is demonstrated that R‐s‐ICG can enrich corneal tissues and pathological blood vessels rapidly with minor residua in normal eyeball tissues. Anti‐CNV treatment‐driven changes in the blood vessels are assessed by real‐time multimodal PAI in vivo, and then a safe laser irradiation strategy through the canthus is developed for phototherapy and gene therapy synergistic treatment. The treatment leads to the efficient inhibition of CNV with faint damages to normal tissues.

## Graphical abstract

A powerful theranostic strategy is presented for noninvasive photoacoustic imaging (PAI)‐guided corneal neovascularization (CNV) treatment. Anti‐CNV treatment‐driven changes in the blood vessels are validated by real‐time multimodal PAI in vivo, and then a safe laser irradiation strategy through the canthus is developed for phototherapy and gene therapy synergistic treatment.

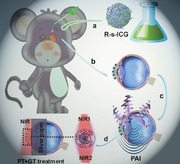

Corneal neovascularization (CNV) has become the second most common cause of blindness all over the world. CNV results from inflammation, viruses, or bacterial infections and trauma to the ocular surface.^[^ ^1^ ^]^ The pathogenesis of CNV results from an imbalance between positive and negative neovascular regulators. Therefore, it is necessary to suppress the formation of CNV while reducing the occurrence of ocular surface lesions.^[^ ^2^ ^]^ Meanwhile, the CNV treatment should not influence the transparency of the corneas. Current CNV treatments include medical treatments and surgical treatments.^[^ ^2d^ ^]^ In medical treatments, topical administration of corticosteroids and nonsteroidal anti‐inflammatory drugs (NSAID) remain the top priority.^[^ ^2e^ ^]^ However, the long‐term administration of drugs may lead to severe side effects like glaucoma, cataract, and superinfection.^[^ ^2f^ ^]^ Surgical treatments mainly include superficial keratectomy, needle diathermy, conjunctival resection, and so on.^[^ ^2g^ ^]^ However, the efficiency of these surgical treatments remains uncertain and depends mainly on the operator's techniques. Thus, it is necessary to develop advance strategies for CNV diagnosis and treatment.

Recently, many imaging techniques have been applied to ophthalmic clinical diagnosis, such as indocyanine green angiography and fluorescein angiography.^[^ ^3^ ^]^ However, the angiography strategy can only show the morphology of blood vessels, and its application in functional imaging‐guided drug delivery remains unsatisfactory. Photoacoustic imaging (PAI) can convert optical absorption/excitation to an ultrasound signal, and this non‐invasive imaging modality is suitable for ophthalmic imaging.^[^ ^4^ ^]^ Other than the standard structural information of tissues, PAI also shows their molecular details for functional imaging, which could provide valuable information about the eyeball and trace medicine.^[^ ^5^ ^]^ For example, PAI could be combined with ultrasound imaging and simultaneously provide anatomical and functional information with increased imaging depth. Furthermore, PAI could be applied to diagnose the oxygen saturation (o.s.) of hemoglobin, which is an essential factor for tissue metabolism.^[^ ^6^ ^]^ Normal corneas have no blood vessels, so PA‐o.s. imaging can be used to observe the development of new blood vessels from CNV. Considering the advantages of PAI, the combined usages of PA‐o.s. imaging and exogenous contrast‐agent‐enhanced PAI make it possible to observe drug accumulation and comprehensively understand the therapeutic effects of this nanomedicine on CNV. Phototherapies (PTs) such as photothermal therapy (PTT) and photodynamic therapy (PDT) have been developed as non‐invasive strategies to treat multiple diseases.^[^ ^7^ ^]^ We intended to construct a PT treatment to deal with corneal diseases through a safety laser irradiation strategy through the canthus.^[^ ^8^ ^]^ However, PT treatment can induce the overexpression of heat shock protein and survivin, which can resist hyperthermia or reactive oxygen species (ROS) damage.^[^ ^9^ ^]^ It is believed that the combination of PT and gene‐therapy (GT) could efficiently eliminate these effects.^[^ ^10^ ^]^ However, the delivery of photosensitizer and gene‐based drugs to corneal blood vessels is challenged by the corneal epithelium and the overlying tear film.^[^ ^11^ ^]^ The intravenous injection of these drugs always requires a large dose, and the consequential negative effects may limit its further application. Conventional methods of eye‐drug administration are eye drops and hydrogel deposits, especially for the anterior segment diseases like CNV. With the development of nanomedicines, the design of the nanodelivery systems might help to verify efficient corneal penetration.^[^ ^12^ ^]^

In a previous study, we reported the preparation of nanoICG theranostics through a self‐assembly procedure between indocyanine green (ICG) and Zn(II)‐dipicolylamine (DPA‐Zn), which were anchored with Bis(DPA‐Zn)‐RGD and survivin‐siRNA (R‐s‐ICG).^[^ ^10a^ ^]^ The R‐s‐ICG nanoplatform exhibited good biocompatibility, outstanding PT property, effective gene transfection, and enhanced angiogenesis‐targeting. We hypothesized that multimodal PAI‐guided regression of CNV might be a key strategy to overcome the difficulties faced in CNV treatment. R‐s‐ICG was administrated to eyes via subconjunctival injections (s.i.) and eye drops (e.d.). This led to nanomedicine accumulations in blood vessels while targeting vascular endothelial cells, as demonstrated by multimodal PAI (PA‐o.s. imaging and ICG‐enhanced PAI). This method enabled the complementary and informative observation of deep tissue in vivo. The e.d. administration of R‐s‐ICG resulted in an acceptable accumulative effect like the s.i. administration strategy, and the R‐s‐ICG e.d. group showed minor residua in normal eyeball tissues. The PT + GT combined treatment was established by a safe laser irradiation strategy through the canthus, and efficient clearance of CNV was achieved with minor damages to normal tissues (**Scheme** **1**). A powerful theranostic strategy is presented for noninvasive PAI‐guided CNV treatment, and a potential system for future clinical translation is suggested.

**Scheme 1:**
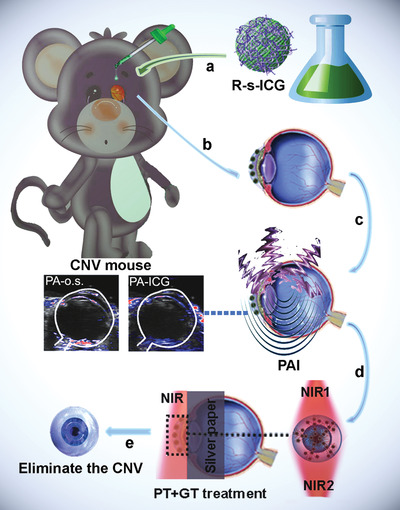
Schematic illustration to the PAI‐guided CNV treatment: a) eye drop (e.d.) of R‐s‐ICG; b) drug accumulation in the CNV tissue; c) PAI monitoring; d) NIR irritation; e) inhibition of CNV.

NanoICG was prepared according to a previous study.^[^ ^10a^ ^]^ The prepared nanoICG comprised nanoparticles of 100–150 nm with good dispersion (Figure S1A, Supporting Information). The nanoICG had unique photo‐properties (Figures S1B and S2, Supporting Information), including fluorescence and PAI properties, enhanced PTT abilities, and acceptable PDT efficacy. For further application, the nanoICG was modified with Bis(DPA‐Zn)‐RGD to prepare modified nanocomposites (R‐nanoICG) with RGD targeting peptide. The modification of RGD could enable R‐nanoICG to target integrin *α*_v_ *β*_3,_ which is over‐expressed exclusively on the vascular endothelial cells of new blood vessels.^[^ ^13^ ^]^ The residual DPA‐Zn could interact with the phosphate radical of siRNA. As shown in Figure S3, Supporting Information, the electrophorectic retardation analysis, dynamic light scattering and zeta‐potential studies confirmed the siRNA loading property of R‐nanoICG. These properties make it possible to use the R‐nanoICG in the PAI‐guided PT and GT treatment of CNV.

Human umbilical vein endothelial cells (HUVECs) were applied in cell studies ex vivo, and corneal endothelial cells (CEnCs) and corneal epithelial cells (CECs) were used as control groups. The expression of survivin was selected as a target for anti‐angiogenesis, and R‐s‐ICG was prepared for the PT + GT combinatorial therapy of HUVECs (**Figure** **1**A). Because the surface of R‐nanoICG was modified with RGD, the R‐nanoICG group showed a stronger fluorescence signal than the ICG and nanoICG groups (Figure S4A, Supporting Information). When CEnCs and CECs were treated with R‐nanoICG, weak fluorescence signals were found (Figure S4B, Supporting Information), indicating the selective uptake ability of HUVECs. As shown in Figure S5, Supporting Information, R‐nanoICG showed no apparent cytotoxicity in HUVECs and the control group, which made it suitable for the further study.

**Figure 1:**
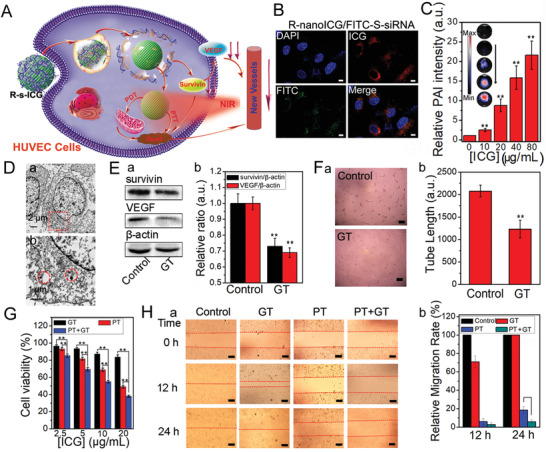
In vitro photo/gene synergistic treatment to HUVECs. A) Schematic illustration to show the combined photo/gene therapy of HUVECs. B) The fluorescence study of R‐nanoICG/FITC‐S‐siRNA in HUVECs. C) The PAI of HUVECs under treatment of R‐nanoICG/FITC‐S‐siRNA. D) Thin‐section cell TEM images of HUVECs incubated with R‐nanoICG/siRNA. E) Western blot (a) and relative intensity (b) of the expression of survivin and VEGF under R‐nanoICG/S‐siRNA treatment. F) The HUVEC tube formation images (a) and tube length (b) under R‐nanoICG/S‐siRNA treatment and the untreated control group. G) Cell viability of HUVECs under the treatment of different concentrations. H) HUVEC migration images (a) and relative migration rate (b) under GT, PTT, PTT + GT treatment for 0, 12, and 24 h. *p< 0.01, **p< 0.05.

To assess the subsequent cell internalization, fluorescein isothiocyanate isomer (FITC)‐labeled S‐siRNA (FITC‐S‐siRNA) was loaded onto the surface of R‐nanoICG. After incubation with HUVECs for 24 h, R‐nanoICG/FITC‐S‐siRNA was exclusively observed in the cytoplasm of HUVECs (Figure 1B). More importantly, S‐siRNA was quickly released from R‐nanoICG, which made the R‐s‐ICG complex suitable for photo/gene combinatorial therapy. The PA signals of HUVECs under treatment with R‐nanoICG/FITC‐S‐siRNA were also studied. As shown in Figure 1C, the PAI intensity increased with the drug concentration and was much higher than that of untreated cells. This provided a possibility for further study in PAI‐guided CNV elimination. Furthermore, cellular TEM data revealed that many of the the nanocomposite was confined in the membrane‐bound intracellular space (Figure 1D).

The amounts of survivin protein and VEGF protein were studied by western blot analysis. As shown in Figure 1E, the expressions of survivin and VEGF were both downregulated in HUVECs treated with R‐s‐ICG. The downregulation of VEGF could prevent the increase of blood vessels, and the downregulation of survivin could promote the apoptosis of HUVECs.^[^ ^14^ ^]^

The tube formation capability of HUVECs under the treatment with R‐s‐ICG was tested by an endothelial tube formation assay. As shown in Figure 1F, few tubes were formed in HUVECs treated with R‐s‐ICG, while plenty of tubes were discovered in the untreated group. Furthermore, HUVEC viability was investigated under different treatments including GT (R‐s‐ICG), PT (R‐nanoICG+NIR), and PT + GT (R‐s‐ICG+NIR). As shown in Figure 1G, PT + GT combinatorial therapy reduced cell viability more efficiently than GT or PT treatment alone.

Next, we examined the migration of HUVECs under different treatments through a scratch‐wound healing assay (Figure 1H). After treatment for 12 h, the cell migration ability of the GT, PT, and PT + GT groups was significantly slowed down in comparison with the wound closure of the control group. In addition, cell migration at 24 h after PT + GT combinatorial treatment was obviously lower than that obtained with PT alone treatment after 24 h. Overall, the PT + GT combinatorial treatment not only killed HUVECs, but also prevented tube formation and migration of HUVECs, suggesting great potential to eliminate new blood vessels.

We investigated a rapid and efficient strategy to treat CNV with R‐s‐ICG. As shown in **Figure** **2**A, we administered R‐s‐ICG in two ways: administration via s.i. in a single dose or administration via e.d. in multiple doses. Firstly, in vivo PA‐o.s. imaging and PA‐ICG imaging of CNV model eyes were compared with untreated eyes. As shown in Figure S6, Supporting Information, no obvious PA‐o.s. or PA‐ICG signals were observed in eyes from the untreated group. However, the eyes of the constructed rat model showed PAI‐o.s. imaging in the cornea, and pathological vascularization could be observed inside the cornea. In addition, the PA‐ICG imaging of the CNV model eyes showed visible signals and could be matched with the PAI‐o.s. imaging. Combined with the PAI property of the prepared nanostructures, the accumulation of R‐s‐ICG in blood vessels could be well studied with the help of PAI‐o.s. imaging and ICG‐enhanced PAI.

**Figure 2:**
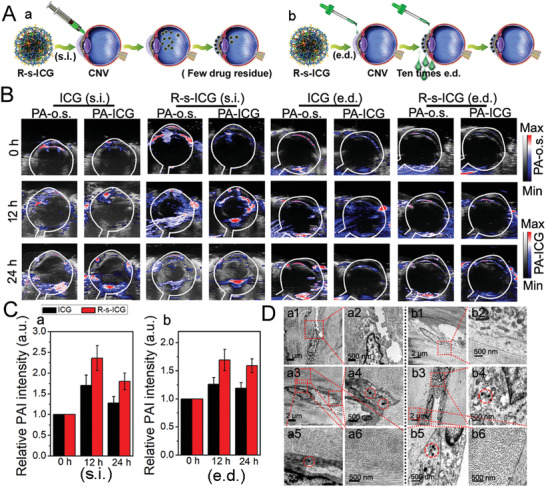
In vivo administration of R‐s‐ICG to corneal neovascularization rat model via subconjunctival injection (s.i.) and eye drops (e.d.). A) Schematic illustration to show the administration of R‐s‐ICG. B) In vivo PA‐o.s. imaging and PA‐ICG imaging of the eyes after administration of R‐s‐ICG. C) Relative PA‐ICG intenstiy of the cornea after subconjunctival injection (a) and eye drops (b) of ICG, and R‐s‐ICG. D) Thin‐section tissue TEM images of eyes after injection of R‐s‐ICG (a1‐5) and the untreated control group (a6), and Thin‐section tissue TEM images of eyes after eye drops of R‐s‐ICG (b1‐5) and the untreated control group (b6).

PA‐o.s. imaging and ICG‐enhanced PAI were next investigated after regular s.i. and e.d. administration of R‐s‐ICG. As shown in Figure 2B, the PA‐o.s. imaging and PA‐ICG imaging of corneas matched with each other very well, implying that PA‐ICG signal changes could be used to study the drug administration of the prepared nanoICG. PA‐ICG signals increased over time after the s.i. and e.d. administration of ICG and R‐s‐ICG. The relative PA‐ICG intensity (Figure 2C) and ex vivo fluorescence of the corneas (Figure S7, Supporting Information) showed that both R‐s‐ICG (s.i.) and R‐s‐ICG (e.d.) were concentrated on the new blood vessels of the corneas. These results demonstrated that R‐s‐ICG had good penetrability in corneal tissues and a targeting effect toward blood vessels. However, the s.i.‐administered R‐s‐ICG not only accumulated in the corneas, but also in vitreous bodies and other eyeball tissues at 24 h post‐injection. It was notable that the R‐s‐ICG (e.d.) group showed minor residua in normal eye tissues at 24 h post‐s.i. administration.

The tissue TEM of R‐s‐ICG‐treated eyeballs (s.i. and e.d.) was also investigated. As shown in Figure 2D, R‐s‐ICG (s.i. and e.d.) accumulated exclusively in the endothelial cells that formed new blood vessels. All these results suggested that R‐s‐ICG (s.i. and e.d.) could be beneficial for PT + GT combinatorial therapy, especially the e.d. administration strategy, in a simple and safe procedure.

To establish the PT treatment, we designed a laser irradiation strategy through the canthus. To avoid retina injury, an 808 nm laser was applied to irradiation from the nasal side to the temporal side of the corneas for 8 min, and then this procedure was repeated in reverse to prevent retina injury (**Figure** **3**A). After R‐s‐ICG (s.i. and e.d.) was accumulated in the new blood vessels of the CNV rat model, the PT + GT combinatorial treatment was performed. We then found that new blood vessels were destroyed and blood flow was blocked. As shown in slit lamp images, the corneal blood vessels of the untreated group increased over time (Figure 3B). After GT treatment (s.i. and e.d.), the increase of corneal blood vessels ceased and decreased to some degree. After PT treatment (s.i. and e.d.), the corneal blood vessels obviously reduced. Compared with GT or PT treatment, the PT + GT combinatorial therapy eliminated corneal blood vessels more effectively. PT + GT (e.d.) also obtained an acceptable therapeutic effect like PT + GT (s.i.).

**Figure 3:**
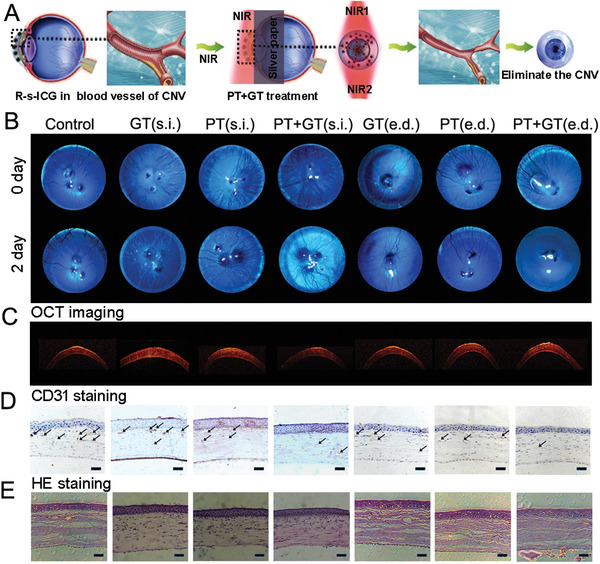
A) Schematic illustration to show the combined photo/gene therapy of corneal neovascularization. B) Slit lamp images of corneal neovascularization model eyes before and after different treatments. C) OCT images, D) CD31 staining, and E) HE staining for corneas after different treatments.

Optical coherence tomography (OCT) was used to study corneal changes under different treatments.^[^ ^15^ ^]^ As shown in Figure 3C, the multilayered structures of the corneas could be noted in OCT images, and no apparent changes were observed in corneal composition. Furthermore, cluster of differentiation 31 (CD31) staining also demonstrated ideal CNV inhibition of the PT + GT combinatorial treatments (s.i. and e.d.) (Figure 3D). Combined with HE staining images (Figure 3E), we concluded that the s.i. and e.d. PT + GT therapy strategies could eliminate most of the corneal blood vessels and inflicted negligible damage to the corneas. One of the advantages of our approach is that it allows the elimination of corneal blood vessels in a short time to avoid the further deterioration in the long‐term treatments (i.e., medical treatments). Apart from this, our strategies provide the easier operation procedures than the surgical treatments, which could be easily handed after a simple training. Thus, our strategies might provide another choice for the clinical CNV treatments.

In summary, we have developed a real‐time in vivo multimodal PAI and photo/gene combinatorial strategy based on nanoICG platforms for the treatment of CNV. The CNV treatment system had several noteworthy features: i) facile tracing of nanomedicine by the combined utilization of PA‐o.s. imaging and the ICG‐enhanced PAI, which could provide the detailed information of drug accumulation to refine the treatment protocol; ii) efficient drug administration to the pathological vessels inside corneas, especially for the e.d. administration strategy, which enables accumulative effects in corneal vessels without residua in normal tissues; and iii) a safe laser irradiation strategy that reduces laser damage to the retina. We believe that the noninvasive PAI‐guided photo/gene combination therapy with the e.d. administration strategy for CNV has important theoretical and clinical significance. However, the in‐depth pharmacokinetic investigations of the nanocomposites in the eyeball and comprehensive safety evaluation of the laser irradiation strategy are needed before the clinic translation.

## 1 Experimental Section

##### In Vitro Cell Study of R‐NanoICG

First, R‐nanoICG was prepared according to the previous study.^[^ ^10a^ ^]^ HUVECs were selected for in vitro cell study. The cytotoxicity of R‐nanoICG was studied by MTT assay. Simply, HUVECs were seeded into 96‐well plates (10^4^ cells/well) for 12 h. Then, R‐nanoICG was added to HUVECs with different ICG concentrations of 6.25, 12.5, 25, 50, and 100 µg mL^−1^, and further cultured for 24 h. At the same time, the control group of CEnCs and CECs were treated under the same condition. All the cells were detected by MTT assay, and the cytotoxicity of R‐nanoICG was determined.

Afterwards, HUVECs were seeded on a glass‐bottomed culture dish at the density of 5 × 10^5^ cells per dish for 12 h. Then, ICG, nanoICG and R‐nanoICG (ICG 20 µg mL^−1^) were added to the culture medium and incubated for 6 h. Besides, the control group of CEnCs and CECs were also seeded on a glass‐bottomed culture dish, and further incubated with R‐nanoICG. Then the culture medium of all the groups were wiped out and washed with PBS, the 4ʹ,6‐diamidino‐2‐phenylindole (DAPI) solution was added, and the fluorescence images of the cells were obtained using a Zeiss confocal microscope.

##### In Vitro Study of HUVECs Under Different Treatments

The survivin‐siRNA (S‐siRNA) was loaded on R‐nanoICG. Simply, 100 µg R‐nanoICG was mixed with 10 pmol S‐siRNA and incubated at room temperature for 30 min to get R‐nanoICG/S‐siRNA mixture (R‐s‐ICG). R‐s‐ICG was studied for further PTT + GT combination therapy of HUVECs. Then, FITC‐S‐siRNA was applied to investigate the cell uptake of R‐nanoICG/siRNA. First, R‐nanoICG and FITC‐siRNA were incubated for 30 min at room temperature. After HUVECs were seeded on a glass‐bottomed culture dish for 12 h, R‐nanoICG/FITC‐S‐siRNA (R‐nanoICG 10 µg mL^−1^) was added to the dish. After incubation for 24 h in the dark, the dish was washed with PBS, and fluorescence images of the cells were obtained using a Zeiss confocal microscope.

The thin‐section cell TEM images of HUVECs incubated with R‐s‐ICG were also studied. Simply, HUVECs were seeded on a glass‐bottomed culture dish for 12 h, and R‐s‐ICG (R‐nanoICG 10 µg mL^−1^) were added to the dish and incubated for 24 h in the dark. After being washed with PBS, the cells were collected for the thin‐section cell TEM image. Western Blot analysis was further applied to study the amount of survivin protein and VEGF protein under R‐s‐ICG treatment. In short, 20 µg mL^−1^ R‐s‐ICG (GT group) was added to the cells and incubated for 48 h in the dark. And untreated cells were set as the control group. Then total proteins of the control group and GT group were harvested, and then the survivin protein and VEGF protein were analyzed by Western Blot.

Furthermore, endothelial tube formation assay was used to study the tube formation ability of HUVECs after GT treatment. Simply, 20 µg mL^−1^ R‐s‐ICG was added to HUVECs and incubated for 36 h in the dark. The untreated HUVECs were set as the control group. Afterwards, the cells were collected and seeded onto Corning Matrigel Basement Membrane Matrix coated 24‐well plates at a density of 10^4^ cells per dish. After incubated for 12 h, the tube structures were observed by using a fluorescent inverted microscope (Nikon Ti‐U). The tube length was analyzed via Image J software.

The PTT + GT combination therapy of HUVECs was further studied. Briefly, R‐nanoICG and R‐s‐ICG (ICG 2.5, 5, 10, and 20 µg mL^−1^) were added to HUVECs and incubated for 24 h in the dark. The untreated cells were set as the control group. After the culture medium was wiped out and washed with PBS, the PTT group and PTT + GT group were exposed to 808 nm laser (1 W cm^−2^) for 8 min. At last, all the cells were further cultured for 24 h and were detected by MTT assay.

The HUVEC migration under different treatments was studied by the wound closure assay. After HUVECs were incubated in 24‐well plate (10^5^ cells per dish) for 12 h, R‐nanoICG and R‐nanoICG/S‐siRNA (ICG 20 µg mL^−1^) were added to HUVECs and incubated for 24 h in the dark. And untreated cells were set as the control group. After the culture medium was wiped out and washed with PBS, a scratch wound was made to each well using a sterile 200 µL pipet tip. For the PTT group and PTT + GT group, the cells were exposed to 808 nm laser (1 W cm^−2^) for 8 min. The wound closure images were obtained using a fluorescent inverted microscope (Nikon Ti‐U) after 1, 3, 12, and 24 h.

##### In Vivo Efficacy for Corneal Neovascularization Rat Model

For the corneal neovascularization study, male Sprague Dawley rats (150–200 g) were obtained from Shanghai SLAC Laboratory Animal Co., Ltd, and raised at Animal Care and Use Committee (CC/ACUCC) of Xiamen University. The rat corneal neovascularization was induced by putting a suture on the central cornea of the left eyes. The animals were observed under the slit lamp microscope, and the corneal neovascularization images were obtained using an Image Pro Plus version 6.0 (Media Cybernetics, Silver Spring, MD, USA).

Ten days after the corneal suturing, new blood vessels were found obviously in rat corneas. Then R‐s‐ICG was administrated via two approaches: subconjunctival injection (s.i.) of nanoformula in one dose and eye drop (e.d.) of nanoformula in multiple doses. i) Administration of R‐s‐ICG (s.i.): 20 µL of R‐s‐ICG nanoformula (1 mg mL^−1^ ICG) was subconjunctivally injected to the rats and fed in the dark. ii) Administration of R‐s‐ICG (e.d.): 5 µL of R‐s‐ICG nanoformula (1 mg mL^−1^ ICG) was topically administrated to the rats and fed in the dark. The topical administration was repeated 10 times every 1 h. The in vivo oxygen saturation and PAI of the eyes were obtained by Vevo 3100 (VisualSonics FujiFilm) at three time points after 0, 12, and 24 h. After that the rats were sacrificed, and the corneas were harvested for tissue TEM study.

For in vivo therapy study, corneal neovascularization model rats were divided into seven groups (*n* = 5): control group, GT group (s.i. and e.d.), PT group (s.i. and e.d.), and PT + GT group (s.i. and e.d.). Then, R‐nanoICG (PT group) and R‐s‐ICG (GT group, PT + GT group) were administrated to the rats and fed in the dark for 24 h. For PTT group and PTT + GT group, the outside NIR irradiation was carried out (Scheme 1): i) first, the eyelid was opened with the eyelid opener, and then the silver paper with a round hole was covered on the CNV eye, thus the cornea was exposed; ii) the laser probe (808 nm) was placed on silver paper, and the distance from the laser to the cornea was 10 mm; iii) the 808 nm laser (0.5 W, 8 min) was irradiated from the nasal side to the temporal side, and then repeated the step in reverse.

After two days, corneal neovascularization of all the rats were observed under slit lamp microscope. Finally, all the rat eyes were harvested. The OCT was applied to study the corneal cross‐sectional thickness and composition. Then CD31 and Hematoxylin and Eosin (H&E) stainings were applied and examined by digital microscopy.

## Conflict of Interest

The authors declare no conflict of interest.

## Supporting information

Supporting Information

## References

[1] a) S. Bochicchio , A. Xhepa , R. Secondi , A. Acquistapace , M. Oldani , M. V. Cigada , A. Giani , G. Staurenghi , Ophthalmol. Retina 2019, 3, 27; 30935656 10.1016/j.oret.2018.08.003

[2] a) Y. Ekim , S. Kara , B. Gencer , T. Karaca , Curr. Eye Res. 2019, 44, 590; 30803276 10.1080/02713683.2019.1584320

[3] a) C. Palme , V. Romano , M. Brunner , R. Vinciguerra , S. B. Kaye , B. Steger , Transl. Vis. Sci. Techn. 2018, 7, 15; 10.1167/tvst.7.5.15 PMC6166904 30280000

[4] a) Y. Liu , P. Bhattarai , Z. Dai , X. Chen , Chem. Soc. Rev. 2019, 48, 2053; 30259015 10.1039/c8cs00618k PMC6437026

[5] a) K. P. Kubelick , E. J. Snider , C. R. Ethier , S. Emelianov , J. Biomed. Opt. 2019, 24, 056004; 10.1117/1.JBO.24.5.056004 PMC6992976 31115200

[6] a) B. Khoobehi , K. Firn , H. Thompson , M. Reinoso , J. Beach , Invest. Ophth. Vis. Sci. 2013, 54, 7103; 10.1167/iovs.13-12723 24114546

[7] a) L. Cheng , C. Wang , L. Feng , K. Yang , Z. Liu , Chem. Rev. 2014, 114, 10869; 25260098 10.1021/cr400532z

[8] a) H. J. Yoon , M. K. Kim , K. Y. Seo , M. Ueta , K. C. Yoon , Int. Ophthalmol. 2019, 39, 55; 29256167 10.1007/s10792-017-0786-x

[9] a) C. Zhang , Y. Yong , L. Song , X. Dong , X. Zhang , X. Liu , Z. Gu , Y. Zhao , Z. Hu , Adv. Healthcare Mater. 2016, 5, 2776; 10.1002/adhm.201600633 27717238

[10] a) C. Chu , E. Ren , Y. Zhang , J. Yu , H. Lin , X. Pang , Y. Zhang , H. Liu , Z. Qin , Y. Cheng , X. Wang , W. Li , X. Kong , X. Chen , G. Liu , Angew. Chem., Int. Ed. 2019, 58, 269; 10.1002/anie.201812482 30421488

[11] a) A. Mukwaya , L. Jensen , B. Peebo , N. Lagali , Ocul. Surf. 2019,17, 400; 30959113 10.1016/j.jtos.2019.04.002

[12] a) H. Han , S. Son , S. Son , N. Kim , J. Y. Yhee , J. H. Lee , J.‐S. Choi , C.‐K. Joo , H. Lee , D. Lee , W. J. Kim , S. H. Kim , I. C. Kwon , H. Kim , K. Kim , Macromol. Biosci. 2016, 16, 1583; 27503770 10.1002/mabi.201600051

[13] J. Wang , W. Li , Z. Lu , L. Zhang , Y. Hu , Q. Li , W. Du , X. Feng , H. Jia , B.‐F. Liu , Nanoscale 2017, 9, 15598. 28990632 10.1039/c7nr04425a

[14] a) X. Fan , N. Gao , J. Li , J. Lei , Q. Kang , Mol. Cell. Biochem. 2018, 441, 173; 28884436 10.1007/s11010-017-3183-x

[15] E. Baghdasaryan , T. C. Tepelus , K. M. Marion , H. Bagherinia , S. R. Sadda , H. Y. Hsu , Cornea 2019, 38, 62. 30211744 10.1097/ICO.0000000000001745

