# Supplementary material for: Multimodal Photoacoustic Imaging‐Guided Regression of Corneal Neovascularization: A Non‐Invasive and Safe Strategy
Source: Adv Sci (Weinh). 2020 May 29;7(14):2000346. doi: 10.1002/advs.202000346 (PMC7375239; doi:10.1002/advs.202000346)
Supplement: Supplementary file 1 — Supporting Information [file ADVS-7-2000346-s001.pdf]

Copyright WILEY-VCH Verlag GmbH & Co. KGaA, 69469 Weinheim, Germany, 2020.

Supporting Information

Multimodal Photoacoustic Imaging-Guided Regression of Corneal Neovascularization: A Non-invasive and Safe Strategy

Chengchao Chu, *† Jingwen Yu,† En Ren,† Shangkun Ou, Yunming Zhang, Yiming Wu, Han Wu, Yang Zhang, Jing Zhu, Qixuan Dai, Xiaoyong Wang, Qingliang Zhao, Wei Li,* Zuguo Liu, Xiaoyuan Chen, and Gang Liu*

Dr. C. Chu, E. Ren, Y. Zhang, Y. Zhang, J. Zhu, Q. Dai, Dr. X. Wang, Dr. Q. Zhao, Prof. G. Liu
State Key Laboratory of Molecular Vaccinology and Molecular Diagnostics & Center for Molecular Imaging and Translational Medicine School of Public Health, Xiamen University Xiamen, 361102, China

Dr. J. Yu, S. Ou, Y. Wu, H. Wu, Prof. W. Li, Prof. Z. Liu
Eye Institute of Xiamen University, Medical College of Xiamen University, Fujian Provincial Key Laboratory of Ophthalmology and Visual Science, Xiamen, Fujian 361102

Dr. X. Chen
Laboratory of Molecular Imaging and Nanomedicine, National Institute of Biomedical Imaging and Bioengineering (NIBIB), National Institutes of Health (NIH), Bethesda, MD 20892, USA

[+] These authors contributed equally to this work.
[*] Corresponding author. Email: gangliu.cmitm@xmu.edu.cn (G.L.); wei1018@xmu.edu.cn (W.L.); chuchengchao0225@163.com (C.C.).

Results and discussion

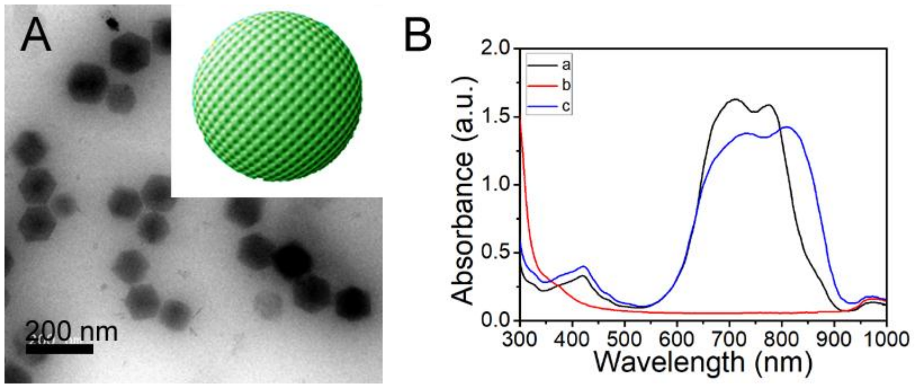

Figure S1 (A) TEM of nanoICG, insert: Schematic illustration to nanoICG. (B) UV-Vis absorption curve of ICG (a), DPA-Zn (b), nanoICG (c).

After the preparation of nanoICG, the UV-Vis of ICG and nanoICG were investigated. And nanoICG possessed absorption of ICG at 650-850 nm (**Figure S1B**), which was suitable for further PT studies.

The photoacoustic (PA) and fluorescence (FL) imaging of nanoICG were further studied. The PA intensity of nanoICG (EX 808 nm) was increased than ICG, which was compatible with most of the assembled ICG nanostructures (**Figure S2A**). And nanoICG showed fluorescence increasing (Ex 710 nm) than ICG (**Figure S2B**), unlike the other aggregation-caused fluorescence quenching.

Furthermore, the PT effect of nanoICG was investigated under 808 nm laser irritation. The temperature of R-nanoICG solution (under 808 nm laser) increased in the initial 4 min and maintained at high temperature in the following 4 min (**Figure S2C**), unlike the unstable PTT property of ICG. Meanwhile, the PDT effect for

R-nanoICG was decreased than ICG (**Figure S2D**), while the weak quenching effect showed no obvious influence on the PDT treatment. All these results implied that nanoICG could be applied in the PAI-guided PTT/PTT (PT) treatment of CNV.

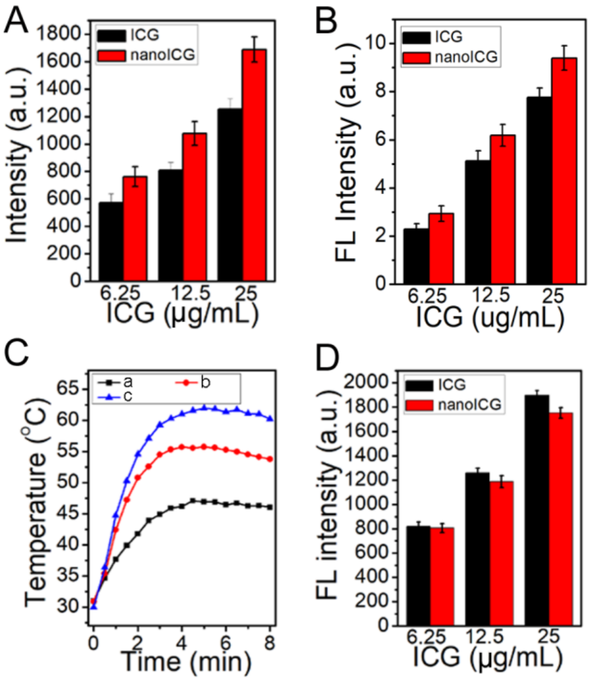

Figure S2 (A) PA values of ICG and nanoICG at EX 808 nm. (B) FL imaging values of ICG and nanoICG at EX 710 nm. (C) Temperatures increase of nanoICG (a 6.25, b 12.5, and c 25 μg/mL) after irradiation under 808 nm laser (1 W/cm^2). (D) ROS production of ICG and nanoICG at EX 808 nm (1 W/cm^2).

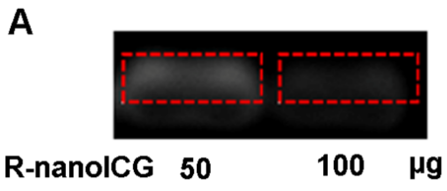

B

	Size (nm)	Potential (mV)
nanoICG	125.4	-23.2
R-nanoICG	131.8	3.7
R-s-ICG	143.2	-5.7

Figure S3 (A) Electrophorectic retardation analysis of siRNA (10 pmol) binding with R-nanoICG. (B) The dynamic light scattering (DLS) and zeta-potential of nanoICG, R-nanoICG and R-s-ICG.

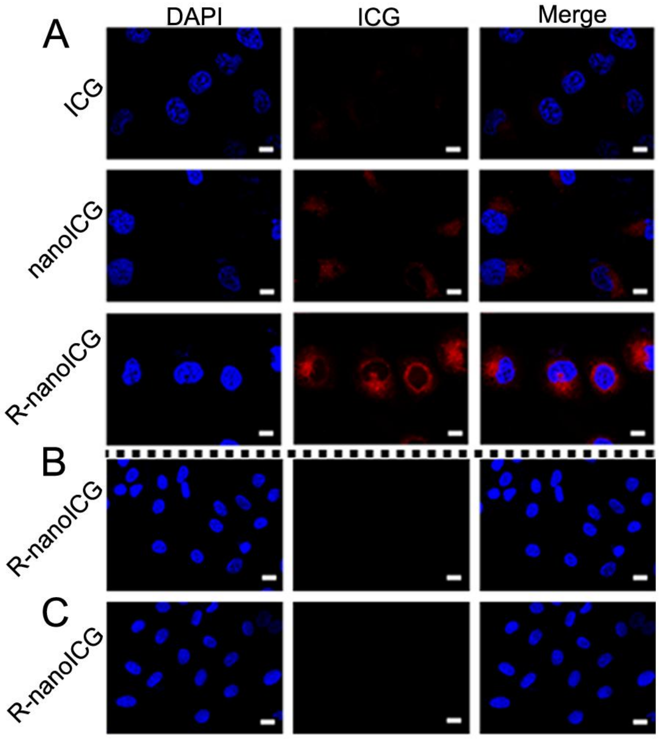

Figure S4 LSCM images of HUVECs under treatment of ICG, nanoICG and R-nanoICG (A), LSCM images of CEnCs (B) and CECs (C) under treatment of R-nanoICG.

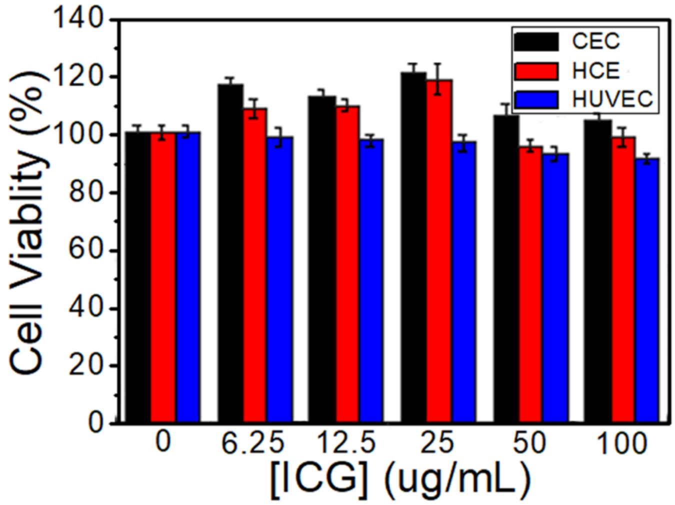

Figure S5 MTT of CEC, HCE and HUVEC cells treated with R-nanoICG.

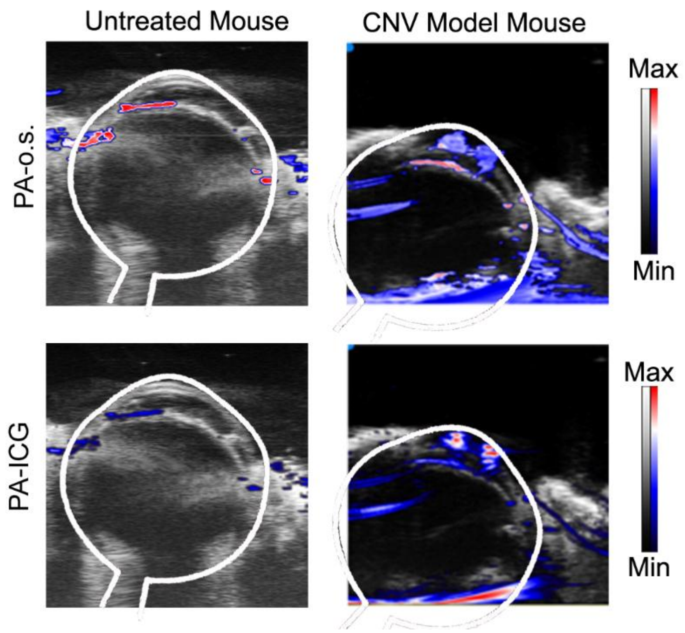

Figure S6 *In vivo* PA-o.s. imaging and PA-ICG imaging of untreated rat eyes and CNV model rat eyes.

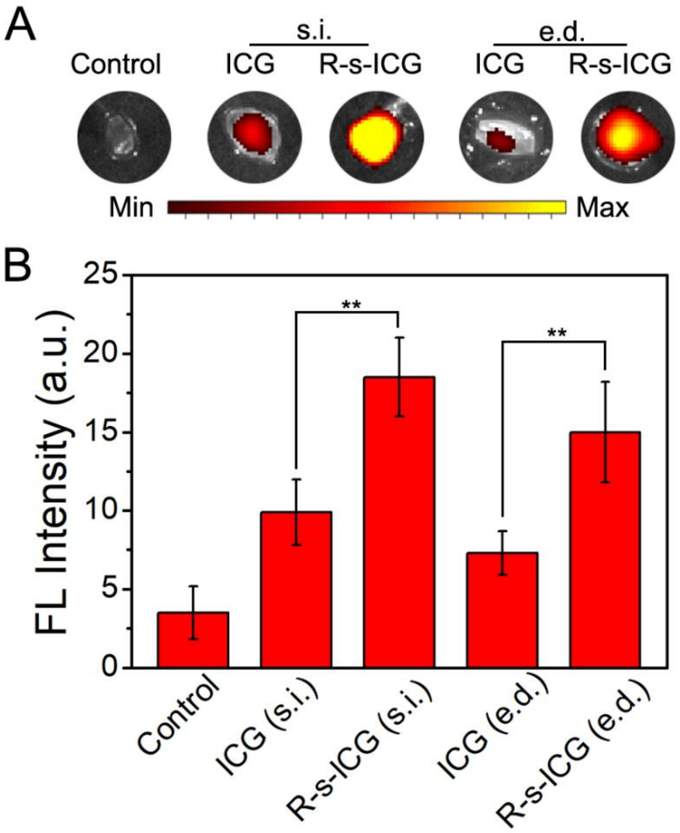

Figure S7 *Ex vivo* near-infrared fluorescence images (A) and relative fluorescence intensities (B) of corneas collected at 24 h post- administration of R-s-nanoICG.
